# CXCR4/SDF1 signalling promotes sensory neuron clustering *in vitro*

**DOI:** 10.1242/bio.035568

**Published:** 2018-08-22

**Authors:** Daniel Terheyden-Keighley, Xiaoqing Zhang, Beate Brand-Saberi, Carsten Theiss

**Affiliations:** 1 Institute of Anatomy, Department of Cytology, Ruhr-University Bochum, Universitätsstraße 150, 44780 Bochum, Germany; 2 Shanghai Tenth People's Hospital, and Neuroregeneration Key Laboratory of Shanghai Universities, Tongji University School of Medicine, 200092 Shanghai, China; 3 Institute of Anatomy, Department of Anatomy and Molecular Embryology, Ruhr-University Bochum, Universitätsstraße 150, 44780 Bochum, Germany

**Keywords:** DRG, Condensation, CXCL12, DSi

## Abstract

During the development of the peripheral nervous system, a subgroup of neural crest cells migrate away from the neural tube and coalesce into clusters of sensory neurons (ganglia). Mechanisms involved in the formation of the dorsal root ganglia (DRG) from neural crest cells are currently unclear. Mice carrying mutations in *Cxcr4*, which is known to control neural crest migration, exhibit malformed DRG. In order to investigate this phenomenon, we modelled sensory neuron differentiation *in vitro* by directing the differentiation of human induced pluripotent stem cells into sensory neurons under SDF1 (agonist), AMD3100 (antagonist) or control conditions. There we could show a marked effect on the clustering activity of the neurons *in vitro*, suggesting that CXCR4 signalling is involved in facilitating DRG condensation.

## Abstract (precis)

**Summary:** The signalling mechanisms directing sensory neuron gangliogenesis are not well understood. Here, we model this process through stem cell differentiation and show that CXCR4 signalling facilitates neural clustering.

## INTRODUCTION

The characterization of molecules that direct the development of the nervous system is vital for advances in neuroregeneration. When looking at the sensory nervous system, sensory neuron cell bodies are organised into clusters adjacent to the spinal cord known as dorsal root ganglia (DRG). The exact signalling mechanisms orchestrating their condensation from neural crest-derived sensory neuron precursors are unknown. Progress on this front has been made examining mutants that display aberrant DRG formation *in vivo* such as a failure to condense into proper ganglia in the *Cxcr4*^−/−^ mouse (Belmadani et al., 2005).

In order to model neurodevelopment *in vitro*, various neuron types have been successfully differentiated from stem cell lines, including midbrain dopaminergic neurons and motor neurons of the spinal cord (Li et al., 2005; Perrier et al., 2004). Differentiation into a (non-placode) sensory neural lineage is more complex due to having first to pass through the transient neural crest stage before reaching the neural precursors and terminally differentiated sensory neurons (Lee et al., 2007). In a 2009 study by Chambers et al., high efficiency neural induction was accomplished by inhibiting both the BMP pathway (transduced via SMAD1/5/8) using noggin, and the TGF-β pathway (transduced via SMAD2/3) using the small molecule SB431542 to block TGFβ1, activin and nodal signalling (Chambers et al., 2009). As this combination inhibits both arms of the internal SMAD signalling pathways (SMAD2/3 versus 1/5/8), it is known as the dual-smad inhibition protocol (DSi).

In a 2012 follow-up study by the same group, the DSi strategy was refined and expanded to rapidly produce nociceptive sensory neurons (Chambers et al., 2012). Based on the observed markers, P2X3^+^, RET^+^ and TrkA^+^, the generated sensory neurons seem to belong to the second non-peptidergic class of sensory neurons (NP2) according to Usoskin et al.’s gene expression clustering classification system (Usoskin et al., 2015). Interestingly, one of the more impressive aspects of this differentiation protocol is its speed; specifically its ability to produce mature nociceptive neurons in just 8–15 days, versus the estimated 30–50 days it takes for a human embryo (Chambers et al., 2012). This makes it a compelling starting point for the modelling of sensory neuron development by allowing us to observe the transient neural crest phase *in vitro*.

One of the signalling systems known to direct neurodevelopment is that of chemokine C-X-C motive receptor 4 (CXCR4) and its exclusive ligand, stromal cell-derived factor 1 (SDF1, also known as CXCL12) (Tamamis and Floudas, 2014). From a central nervous system perspective, CXCR4 is constitutively expressed in all major cell types of the brain including neurons, astrocytes and microglia (Lazarini et al., 2003). There it has been shown to be vital for the normal development of the hippocampus and cerebellum (Lu et al., 2002; Zhu et al., 2002). In the peripheral nervous system (PNS), neural movement due to CXCR4/SDF1 signalling during development can be divided into two categories: whole cell migration and axon growth cone guidance. CXCR4 signalling has been shown to direct the initial neurite outgrowth direction of motor neurons in the spinal cord and guide sensory neuron innervation through the dorsal root entry zone (Chalasani et al., 2003; Lieberam et al., 2005). In terms of cellular migration, neural crest cells have been shown to follow cues of CXCR4 signalling to differentiate DRG precursors from sympathetic ganglia precursors (Kasemeier-Kulesa et al., 2010). Finally, the *Cxcr4*-null mouse shows small fragmented DRG based on nociceptor cell staining, indicating a possible role directing their formation (Belmadani et al., 2005).

Based upon these previously reported effects on mouse DRG formation, we modelled the development of human nociceptors *in vitro* and examined the effects of SDF1 (agonist) and AMD3100 (inhibitor) on both their differentiation and morphology. The *in vitro* model is based on the directed differentiation of human induced pluripotent stem (hiPS) cells into nociceptive sensory neurons using a range of inhibitors of key pathways at specific time points. This study follows their differentiation through a transient neural crest phase by screening for developmental marker genes over multiple time points using qPCR and immunohistochemistry. Here we could observe differences in cell clustering behaviour between agonistic and inhibitory conditions.

## RESULTS AND DISCUSSION

With the CXCR4-homozygous mutant mouse showing ectopically located TrkA^+^ nociceptive neurons in malformed ganglia fragments (Belmadani et al., 2005), we set up an *in vitro* model of differentiating sensory neurons from human induced pluripotent stem cells (hiPSCs). Here our goal was to test whether CXCR4 signalling affected the differentiation of sensory neurons *in vitro*, and to see if there was an alteration in their morphology. We used the DSi protocol (Chambers et al., 2009) to first drive neural differentiation, followed by a modified patterning protocol based on three inhibitors and agonists of key embryonic pathways to efficiently drive a TrkA^+^ nociceptor sensory neuron fate (Chambers et al., 2012). Our modifications included seeding density, patterning-factor timing and general media optimisations. The cultures were split into three groups; control (*N*=6), medium+SDF1 (agonist; *N*=6) or medium+AMD3100 (inhibitor; *N*=6), each added from the second day of differentiation onwards. These groups were multiplied by the number of time points for fixation and analysis (Day 4, 8, 12 and 15) and cultured in parallel.

### *In vitro* sensory neuron differentiation models neural crest transition

**Fig. 1.**
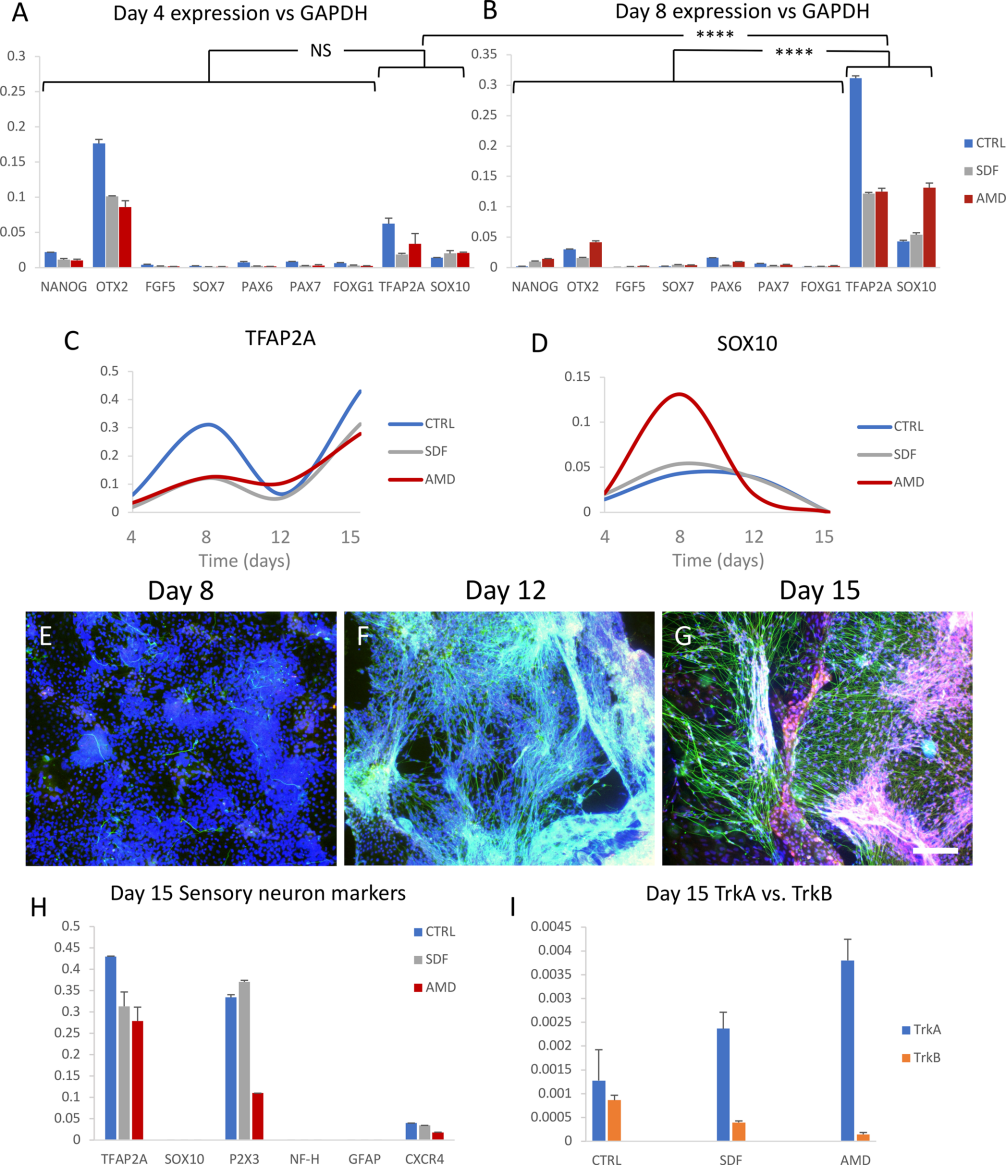
**Directed differentiation of hiPSCs to sensory neurons under CXCR4 stimulation or inhibition.** Differentiation cultures from four time-points (4, 8, 12 and 15 days) were cultured, each with three groups [control, inhibitor (AMD3100) and agonist (SDF1)] with six experimental replications each (72 cultures total). (A,B) Transcription level of various markers relative to GAPDH in the three differentiation groups on days 4 and 8 shows the transition to neural crest cells (TFAP2A/SOX10). Grouping neural crest markers shows their expression to be not significantly (NS) higher than non-neural crest markers on day 4, but highly significant (****P<0.0001) by day 8. (C,D) expression of TFAP2A and SOX10 in the three groups relative to GAPDH over four time-points (day 4, 8, 12 and 15). (E–G) Sensory neuron differentiation progression of the control group with nuclei in blue, neurites (beta-III-tubulin) in green, and TrkA in red (sensory neurons, G only) on days 8, 12 and 15. (H) Transcription levels of markers relevant to sensory neuron differentiation and subtype specification relative to GAPDH on day 15. (I) Transcription levels of TrkA and TrkB in the three groups relative to GAPDH on day 15. qPCR: single cDNA pool from two replications, error bars: standard deviation of qPCR replications. Scale bar: 150 µm.

Immunostaining and qPCR were used to monitor the differentiation progress of the differentiating iPS cells by looking at a variety of lineage markers (Fig. 1A,B). Specifically, around the middle of the differentiation, we can see an upregulation of *Sox10* and *Tfap2a*, markers of the neural crest, suggesting that the cultures are transitioning through this transient cell type the same as *in vivo.* Comparing the transcription levels of neural crest verses non-neural crest markers shows a highly significant increase of these markers by day 8 (Fig. 1C,D). The abrupt rise in *Tfap2a* seen by day 15 is in line with the formation of neural crest-derived organs, presumably sensory ganglia in this case (Mitchell et al., 1991). *Sox10* expression also corresponds with neural crest specification, however, more telling is how it is almost completely extinguished in all three groups by day 15, indicating the end of the transition from neural crest into sensory neurons. This is based on observations that *Sox10* expression is also extinguished *in vivo* when progenitor cells in the outer layer of the developing DRG migrate towards the core while differentiating towards the neural lineage (Sonnenberg-Riethmacher et al., 2001). DRG cells that are destined to become glia such as Schwann cells or satellite glia maintain *Sox10* expression, and so the lack of it by day 15, along with the lack of *Gfap* expression indicates the absence of glial differentiation in this model (Britsch et al., 2001).

*Otx2* is highly expressed on day 4 (Fig. 1A) and is a marker for anterior neural plate identity (Acampora et al., 1995). However as *Foxg1* expression remains negligible, a possible subpopulation of ventral telencephalon neurons is ruled out (Martynoga et al., 2005). *Otx2* is also implicated in epiblast cells, suggesting this to be the transient cell type (Tesar et al., 2007). The expression of other non-neural crest markers was negligible during this time, indicating efficient specification with very little off-target differentiation. This includes the pluripotency marker, *Nanog* (Mitsui et al., 2003), primitive streak epiblast marker, *Fgf5* (Hebert et al., 1991), extra embryonic endoderm marker, *Sox7* (Kanai-Azuma et al., 2002) and neural plate marker, *Pax6* (Pevny et al., 1998; Zhang et al., 2010). The higher variability between differentiation groups prior to day 15 is expected given the low temporal resolution of four-day intervals between measurements of differentiating cell cultures. We consider them a rough guide for tracking differentiation progress, however, as *Sox10* expression is extinguished by day 15 in all groups, we take that to be the end of differentiation and thus markers from day 15 as more relevant for comparison purposes. The qPCR tracking data suggests there are no differences between groups on day 15 when looking at the neural crest markers, *Sox10* or *Tfap2a* (Fig. 1C,D); however, more data is needed to confirm this.

Based on the expression of the mature neural marker, beta-III-tubulin, the first mature neurons could be seen at day 8 in control cultures, followed by the rapid emergence of a dense neurite meshwork by day 12 (Fig. 1E,F). The start of terminal differentiation (first expression of mature neural marker beta-III-tubulin) for all three groups was approximately synchronized with comparable cell densities (Fig. S1). Staining for TrkA on day 15 revealed predominantly nociceptor neurons (Fig. 1G, see Fig. S2 for antibody positive controls), in accordance with Chambers et al.’s differentiation protocol, which also achieved a 78% nociceptor fate (Chambers et al., 2012). TrkB staining was negligible, indicating we are mainly generating nociceptive neurons, as opposed to mechanoreceptive sensory neurons.

On day 15, additional markers were used to look at sensory neuron subtype specification (Fig. 1H). The high levels of *P2X3*, an ATP-evoked nociceptor activation receptor, match Chambers et al.’s characterisation of the differentiation protocol (Chambers et al., 2012; Lewis et al., 1995). The TrkA expression points towards the NP2 sub-class of nociceptor according to Usoskin et al.’s classification system (Usoskin et al., 2015). This fate is supported by the lack of neurofilament heavy chain (*Nf*-*h*), suggesting an absence of myelinated TrkB^+^ or TrkC^+^ sensory neurons.

**Fig. 2.**
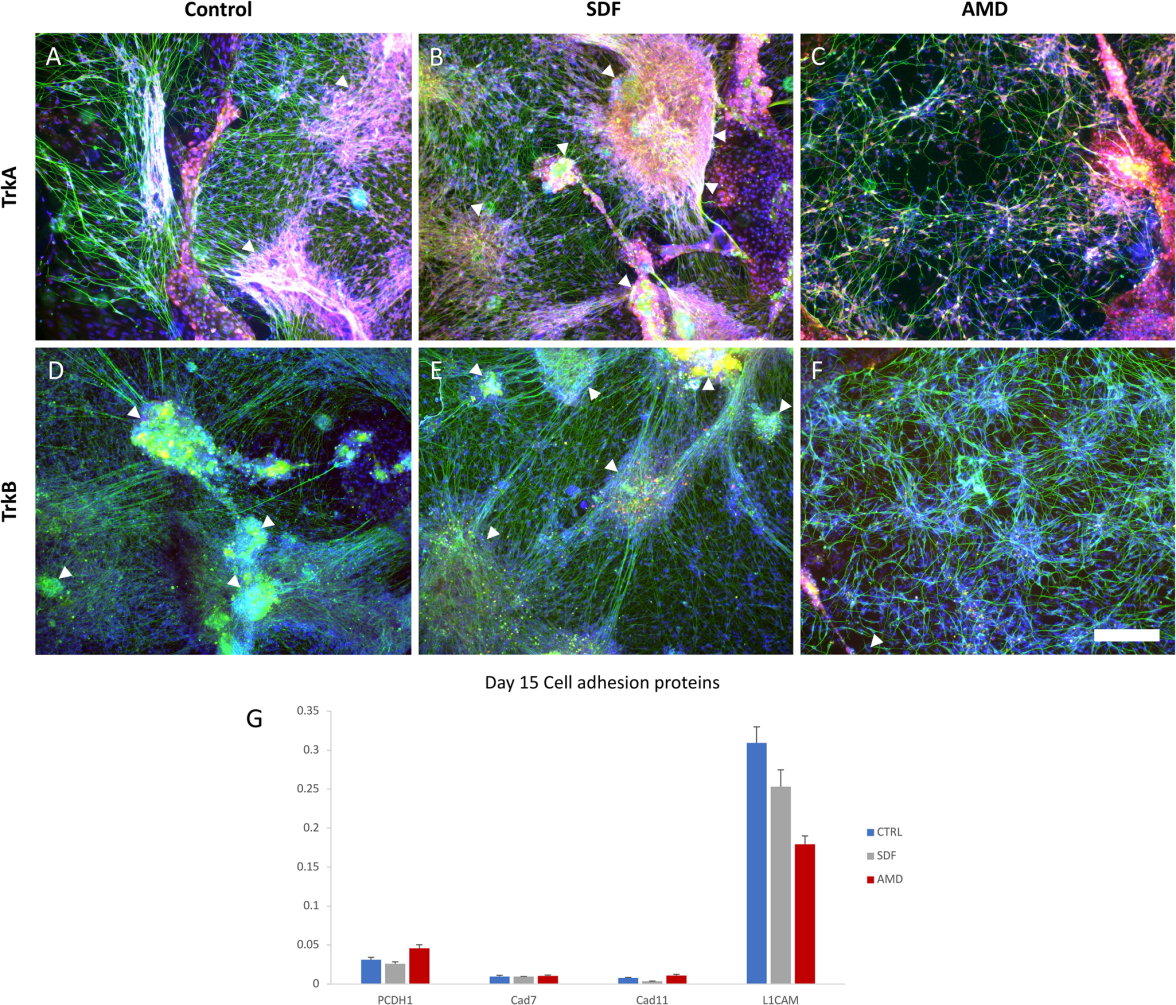
**Culture morphology and cell adhesion molecule expression analysis after CXCR4 stimulation or inhibition.** (A,B,C) Day 15 cultures from the control, SDF1 (agonist) and AMD3100 (antagonist) groups stained for TrkA (red, nociceptive marker), nuclei (blue) and beta-III-tubulin (green, neurite marker). (D,E,F) Day 15 cultures from the Control, SDF1 and AMD3100 groups stained for TrkB (red, mechanoreceptive marker), nuceli (blue) and beta-III-tubulin (green, neurite marker). Control (with endogenous SDF1) and SDF1-stimulated differentiation cultures develop into DRG-like clusters of radially projecting nociceptive neurons (arrows), whereas inhibitory conditions result in a more evenly distributed phenotype when looking at beta-III-tubulin staining (*N*=3 per condition, none excluded). (G) Comparison of transcript levels of various cell adhesion proteins between the differentiation groups relative to GAPDH (Single cDNA pool from two replications). Error bars: standard deviation of qPCR replications. Scale bar: 150 µm.

When examining the distribution of *TrkA* and *TrkB* transcripts on day 15 (Fig. 1I), they seem to show a bias towards *TrkA^+^* sensory neuron specification when interfering with CXCR4/SDF1 signalling, however due to the very low total transcript amounts relative to GAPDH (1000x lower), no conclusions were drawn. The distribution also disagrees with the immunostaining data, as TrkA immunoreactivity greatly outweighs that of TrkB (Fig. 2A–D); however, as down regulation at the protein level lags behind that of transcriptional activity, the neurons may be in the process of transitioning to a TrkA^−^ non-peptidergic subtype such as NP1 or NP3 (Chambers et al., 2012). This is supported by the observation that all nociceptive neurons start out as TrkA^+^, with certain subpopulations switching from NGF-dependent survival to GDNF-dependent Ret^+^ (GDNF receptor) nociceptors over time (Chen et al., 2006).

### CXCR4 signalling promotes ganglia-like condensation *in vitro*

When looking at sensory neuron subtype specification via TrkA/B immunostaining, no obvious differences could be seen in their expression relative to one another when comparing CXCR4 inhibitor and agonist conditions. The majority stained positive for TrkA, while virtually none stained positive for TrkB (Fig. 2A–F). However, when examining the clustering behaviour of these two groups, there does appear to be a clear difference, with ganglia-like clusters forming under SDF1 and control conditions, whereas a more loosely distributed meshwork of neurons can be seen under inhibitory conditions (Fig. 2, see Fig. S3 for large 6×6-image slide scans of additional cultures, and Fig. S4 for separate colour channels). This would suggest that these cells are secreting SDF1 themselves, else we would not expect to see a difference between control and inhibitory conditions, and indeed, low levels of transcription were detected.

Other than clustering behaviour, differences are apparent in neurite density, axon tension and fasciculation. These processes are facilitated by cell adhesion proteins, and so we checked for differences in their relative expression levels between differentiation groups (Fig. 2G). These include proto cadherin 1 (PTCDH1), cadherin 7 (Cad7) and 11 (Cad11), and finally the neural cell adhesion molecule, L1CAM. The cellular adhesion molecules thought to be responsible for sorting sensory progenitor cells and organizing the structure of the developing DRG are the cadherins (Bononi et al., 2008; Chalpe et al., 2010). The delamination and migration of neural crest cells from the neural tube were shown to be based on the regulation of cadherin 7 and 11 by canonical Wnt signalling (a prominent player in neural crest specification) (Chalpe et al., 2010). The manipulation of protocadherin-1 or SDF1 both result in a shift in the distribution of cells between the sympathetic ganglia and DRG (Bononi et al., 2008; Kasemeier-Kulesa et al., 2010). CXCR4 signalling may thus be acting as a permissive factor for cell adhesion, facilitating the sensory precursors coalescing during ganglia condensation.

At a transcriptional level, none of the cellular adhesion molecules exhibited changes large enough to explain the complete lack of clustering seen under inhibitory conditions (AMD3100). L1CAM has been shown to be highly expressed on DRG nociceptive afferents innervating the spinal cord, and as such, the high levels detected in our cultures provides further evidence of a predominant nociceptor fate (Runyan et al., 2005). L1CAM is additionally heavily regulated at the post-transcriptional level by being trafficked to the cell membrane and by the presence of sialic acid, and so cannot be ruled out as a mediator of the observed clustering from qPCR data alone (Hoffman and Edelman, 1983; Yamanaka et al., 2007). Overall this suggests that CXCR4 signalling plays a role in neural clustering *in vitro*, however not via modulating the transcription of these adhesion proteins.

In conclusion, to test whether the CXCR4/SDF1 signalling axis affects the differentiation of sensory neurons, we used an *in vitro* sensory neuron differentiation method which modelled the transient neural crest stage. When examining the morphology of these cultures, we could see differences in clustering behaviour, suggesting that our differentiated sensory neurons may represent nociceptors that require CXCR4 signalling for condensation. Transdifferentiation models such as the one used here represent powerful methods for studying transient cell types such as neural crest *in vitro*, especially when it comes to migration. Future CXCR4 experiments should focus on its molecular mechanisms in cell clustering and migration, along with experiments examining cellular adhesion molecules at the protein level in order to further assess CXCR4's impact on PNS development.

## MATERIALS AND METHODS

### hiPSC culture

hiPSC [Passage number 26–35, WiCell Institute, Wisconsin University, lines described by Hu et al. (2010)] were kept in culture on irradiated mouse embryonic fibroblast (MEF) feeder cells in ES medium [20% KOSR (125 ml, 10828-028, Gibco), 3.125 ml glutamax (35050-061, Gibco), 6.25 ml NEAA (11140-050, Gibco), 4.5µl beta-mercaptoethanol (0482, Amresco, Solon, USA) in 500 ml DMEM/F12 (11330-032, Gibco)] with an additional 4 ng/ml of bFGF (13256029, Invitrogen) at 37°C and 5% CO_2_. The medium was changed daily. The cultures were passaged when colonies had reached a large diameter, before signs of differentiation were visible (poorly defined boarders/non-uniform colour). Passaging entailed first washing with DMEM (11965-092, Gibco), then digestion with 1 ml pre-warmed dispase (17105, Gibco) for 3–5 min at 37°C. The dispase was then carefully aspirated, before the cells were collected in ES medium via mechanical dislocation and trituration with a pipette tip. The collected ES colonies were then further triturated in a 50 ml tube to obtain cell clusters of the appropriate size. These clusters were then centrifuged for 1 min at 200×***g*** RCF, before being resuspended in ES media+4 ng/ml bFGF at the appropriate concentration (typically 1:6 split). The clusters were finally seeded onto MEF which had been pre-rinsed with DMEM.

### Sensory neuron differentiation

Human iPS cells were differentiated into primarily nociceptive neurons using a modified dual-smad inhibition protocol (Chambers et al., 2012). In brief, hiPSC colonies were treated with 10 mM ROCK inhibitor (Y-27632, SCM075, EMD Millipore) 3 h before being washed with DMEM and then digested into single cells by incubation with accutase (A11105-02, Gibco) at 37°C for 20 min. The cells were collected in a 5× volume of ES medium and triturated with a pipette to mechanically disassociate any clumps. The cell suspension was centrifuged at 450×***g*** RCF for 2 min before resuspension in ES medium+4 ng/ml bFGF+10 mM ROCK inhibitor at the correct cell density for seeding. The cells were seeded in this medium at 40,000 cells/cm^2^ onto Matrigel-coated glass coverslips [1 h incubation with 1:50 Matrigel (354234, Corning, New York, USA) in DMEM, aspirated at time of seeding] and left to adhere overnight at 37°C and 5% CO_2_. The next day marks day 0, where the medium was exchanged for ES medium+DSi [10 µM SB431542 (04-0010, StemGent, Beltsville, USA) and 100 nM LDN193189 (04-0074, StemGent)]. Day 1: media change. Day 2: medium changed with ES medium+DSi+i3 [5 µM SU5402 (S7667, Selleck, Houston, USA), 10 µM DAPT (S2215, Selleck) and 3 µM CHIR99021 (S2924, Selleck)]. Day 4: 75% ES medium+25% N2 medium [1× N2 (17502-048, Gibco) +1× B27 (12587-010, Gibco) in neurobasal medium (21103-049, Gibco)] +DSi+i3. Day 6: 50% ES medium+50% N2 medium+i3. Day 8: 25% ES medium+75% N2 medium+i3+3NT [50 ng/ml NGF (#SRP3015, Sigma-Aldrich), 20 ng/ml BDNF (450-02, Pepro Tech, Rocky Hill, USA) and 20 ng/ml GDNF (450-10, Pepro Tech)]. Day 10: N2 medium+i3+3NT. From here on the medium was changed every second day. 20 µM AMD3100 (S8030, Selleck) or 100 ng/ml SDF1 (#300-28A, Pepro Tech) was added to the media of their respective test groups from day 2 onwards.

### RNA extraction

For qPCR, RNA was first extracted from the differentiating cells at room temperature, pooling two of the six wells per differentiation group. First the cells were incubated in Trizol (15596026, Ambion, Carlsbad, USA) for 5 min, before scraping/collecting the cells in a pipette and transferring them into chloroform [(0705, Ambion) 1:5 chloroform:Trizol]. This was shaken vigorously for a few seconds before being left to stand for 5 min. Next the solution was centrifuged in a microfuge at max speed (13 K RPM) for 10 min, after which the top phase was transferred into 2-propanol [(A451-4, Fisher Chemical, Geel, Belgium) half the volume compared to Trizol] and mixed again. This was left to precipitate at −80°C for 10 min, before being centrifuged again at max speed for 15 min to pellet the RNA. The pellet was then washed in 75% ethanol (G73537B, General-Reagent, Shanghai, China), centrifuged at max speed for 5 min, left to air-dry and then resuspended in 10 µl of RNase-free water. RNA concentration was determined via nanodrop (2000C, Thermo Fisher Scientific).

### Reverse transcription

For reverse transcription, 1 µg of RNA was mixed with 1 µl dNTP (4019, Takara, 10 mM) +1 µl random primer (51709, Invitrogen, 50 ng/µl) and brought up to 10 µl with RNase-free ddH_2_O. This was then incubated at 65°C for 5 min, before adding 4 µl of 5× RT buffer (Y02321, Invitrogen), 2 µl of 0.1 M DDT (Y00147, Invitrogen, 0.1 M), 0.25 µl RNase OUT (100000840, Invitrogen, 40 U/µl), 0.25 µl Script III (56575, Invitrogen, 200 U/µl) and 3.5 µl RNase-free ddH_2_O for a total of 20 µl. A thermal cycler was then used to incubate the solution at 25°C for 10 min, then 50°C for 50 min and finally 85°C for 5 min. The 20 µl cDNA was diluted with 380 µl H_2_O to be used as a qPCR template.

### qPCR

Primers for a range of developmental marker genes (Table S1) were used in qPCR to identify the differentiation state of the cell cultures. Per reaction, 0.5 µl forward primer, 0.5 µl reverse primer, 10 µl 2× qPCR mix (RR820, Takara), 5 µl H_2_O and 4 µl template were mixed together in 96-well plates (HSP9601, Bio-Rad) before being centrifuged at 1500×***g*** RCF for 1 min. qPCR was performed in a light cycler (788BR01128, Bio-Rad) using an annealing temperature of 55°C (program: 95°C for 5 min, 39 cycles of 95°C, 55°C then 72°C for 30 s each, and finally 72°C for 5 min. Melt curve: 65°C to 95°C increment 0.5°C for 5 s+plate read). All reactions were run as duplicates, with the average normalised CT values used to obtain a gene's relative expression level verses GAPDH.

### Statistical methods

To assess whether the differentiating sensory neurons transitioned through the neural crest lineage, a two-way ANOVA was performed after grouping the transcription levels of the neural crest marker genes (TFAP2A and SOX10) and non-neural crest markers (the rest). Comparisons made between time points illustrate whether a significant difference exists in expression level of neural crest groups, also using a two-way ANOVA. The confidence interval for highly significant results is *P*<0.0001 (****).

### Immunostaining

Cell cultures on the glass coverslips were rinsed with PBS before being fixed in 4% PFA for 10 min. Following this, the coverslips were briefly rinsed twice in PBS, then washed twice for 5 min in PBS at room temperature. Next the cells were blocked and permeabilised in blocking buffer [0.1% triton-X-100, 10% donkey serum (017-000-121, Jackson ImmunoResearch, 60 mg/ml) in PBS] for 1 h at room temperature. Primary antibodies (Table S2) were applied at the appropriate concentration in 50% blocking buffer in PBS overnight at 4°C. The following day, the coverslips were fully washed (2× rinse in PBS, followed by 3×10 min washes in PBS at room temperature). Then the coverslips were incubated with the appropriate secondary antibodies (1:1000) for 1 h at room temperature [red anti-rabbit (711-165-152, Jackson ImmunoResearch, 1.5 mg/ml), green anti-mouse (715-485-150, Jackson ImmunoResearch, 1.4 mg/ml), far red anti-rabbit (211-602-171, Jackson ImmunoResearch), red anti-goat (705-165-147, Jackson ImmunoResearch, 1.5 mg/ml)]. After another full wash step, Hoechst solution (5824, Tocris, 1:1000) was applied for 10 min, followed by a full wash step. The coverslip was then finally mounted onto a glass slide in mounting medium (H-1000, Vector, Burlingame, USA). Imaging was performed using a Nikon spinning disc confocal microscope (CSU-W1, Visitron Systems, Puchheim, Germany) with its PlanApo 10× (NA 0.25) and 20× (NA 0.75) objectives.

## Supplementary Material

Supplementary information
