# Supplementary material for: CXCR4/SDF1 signalling promotes sensory neuron clustering in vitro
Source: Biol Open. 2018 Aug 22;7(9):bio035568. doi: 10.1242/bio.035568 (PMC6176946; doi:10.1242/bio.035568)
Supplement: Supplementary information [file biolopen-7-035568-s1.pdf]

Supplementary

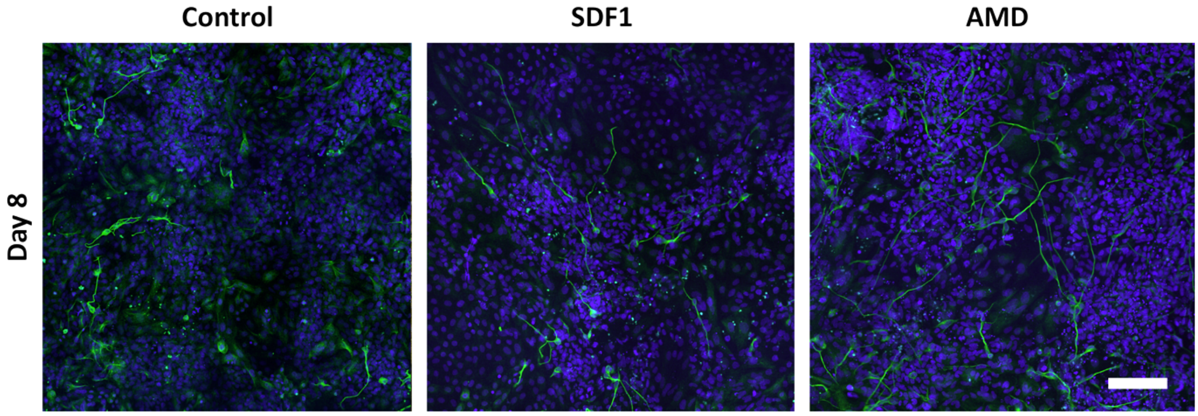

Figure S1 – shows day 8 of the sensory neuron differentiation protocol for the three groups: control, SDF1 and AMD3100. Cultures stained for beta-3-tubulin (green) and exhibit a similar onset point of terminal differentiation, whereas nuclear staining (blue, Hoechst) reveal comparable cell densities across groups. Scale bar: 150μm.

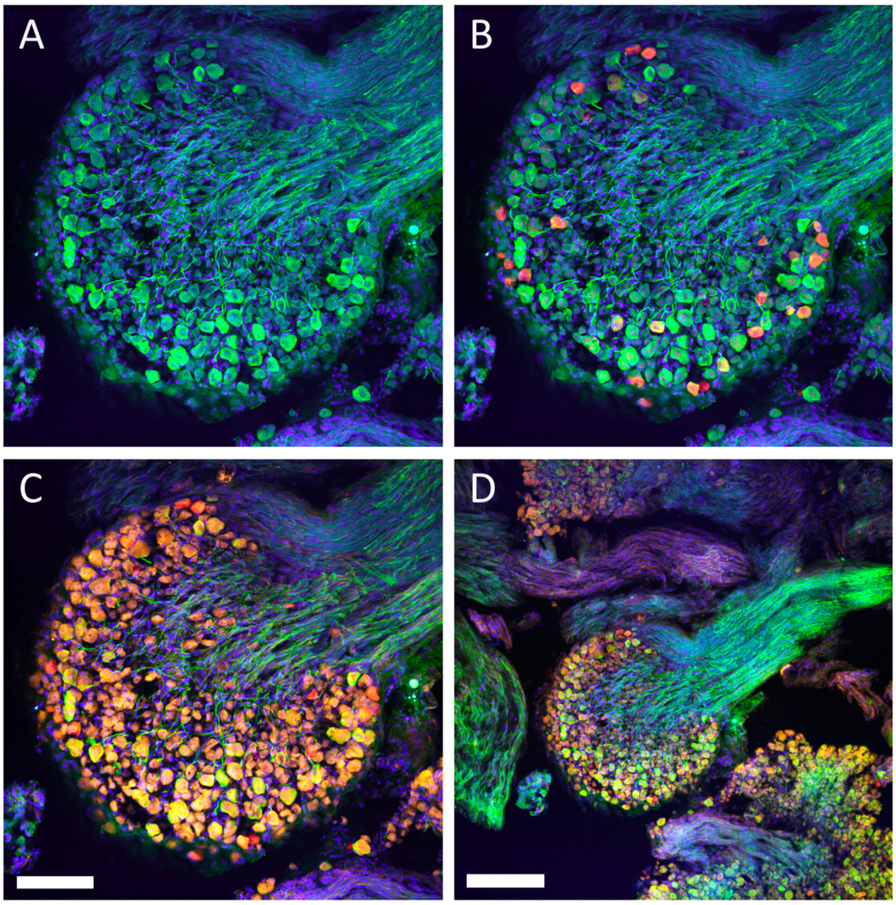

Figure S2 – shows immunostaining antibody positive control. Cryosectioned (50μm) post-natal day 6 mouse DRG immunostained against beta-III-tubulin (green), TrkA (yellow) and TrkB (red), with nuclei stained blue using hoechst. A) Hoechst + beta-III-tubulin (20x obj.) B) Hoechst + beta-III-tubulin + TrkB (20x obj.) C) Hoechst + beta-III-tubulin + TrkB + TrkA (20x obj. – Scale bar: 150μm) D) Hoechst + beta-III-tubulin + TrkB + TrkA (10x obj. – Scale bar: 300μm).

Biology Open • Supplementary information

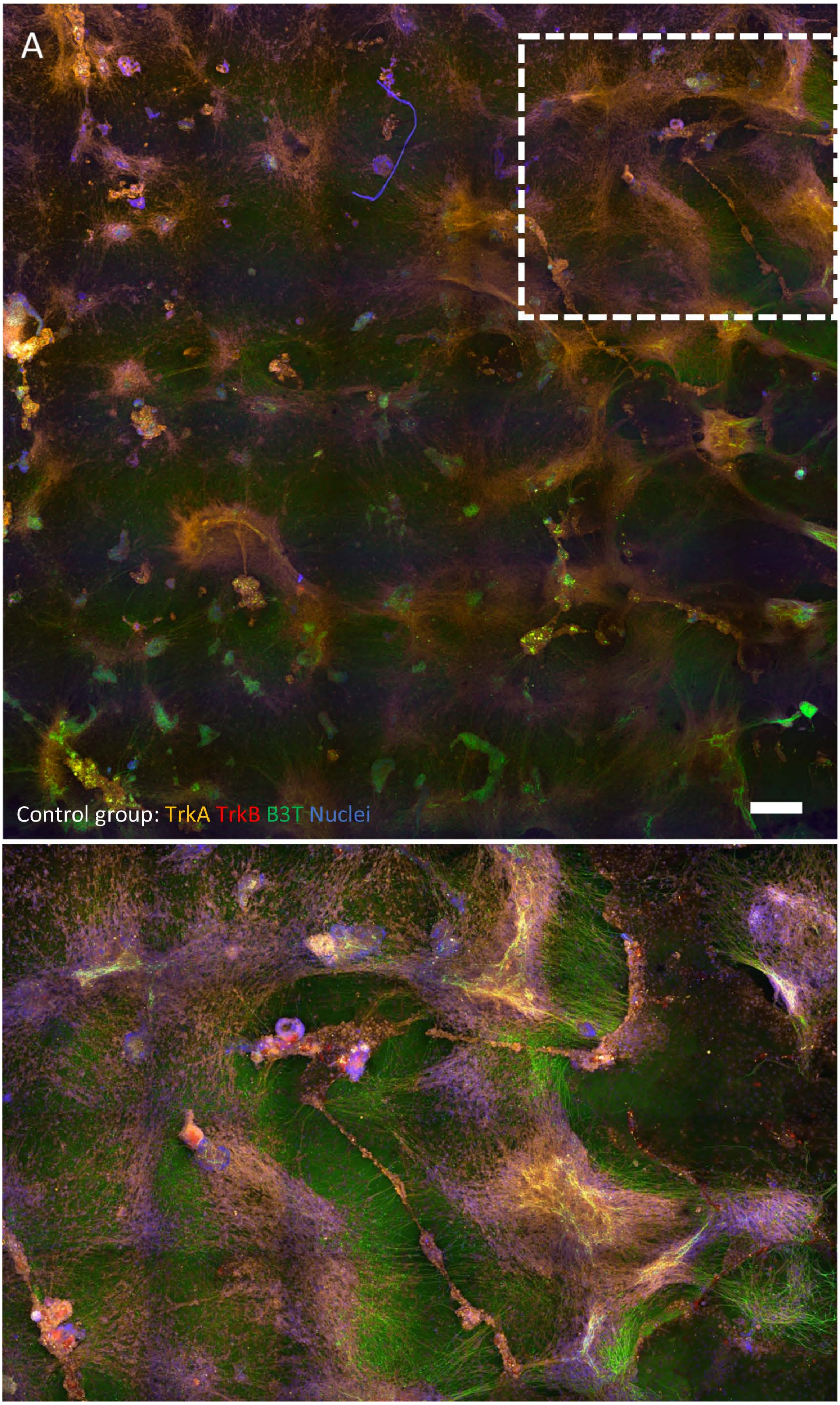

Biology Open • Supplementary information

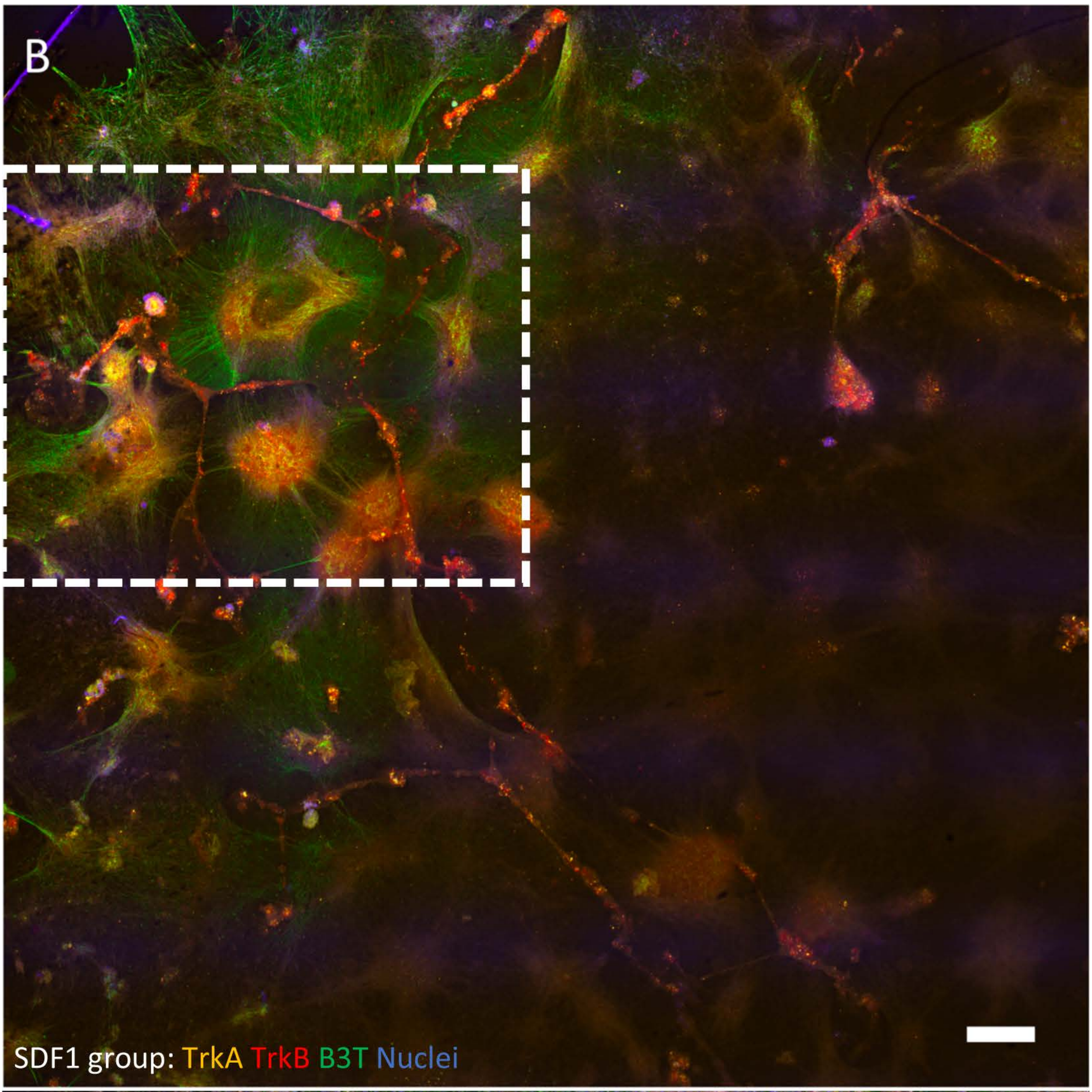
B
SDF1 group: TrkA TrkB B3T Nuclei

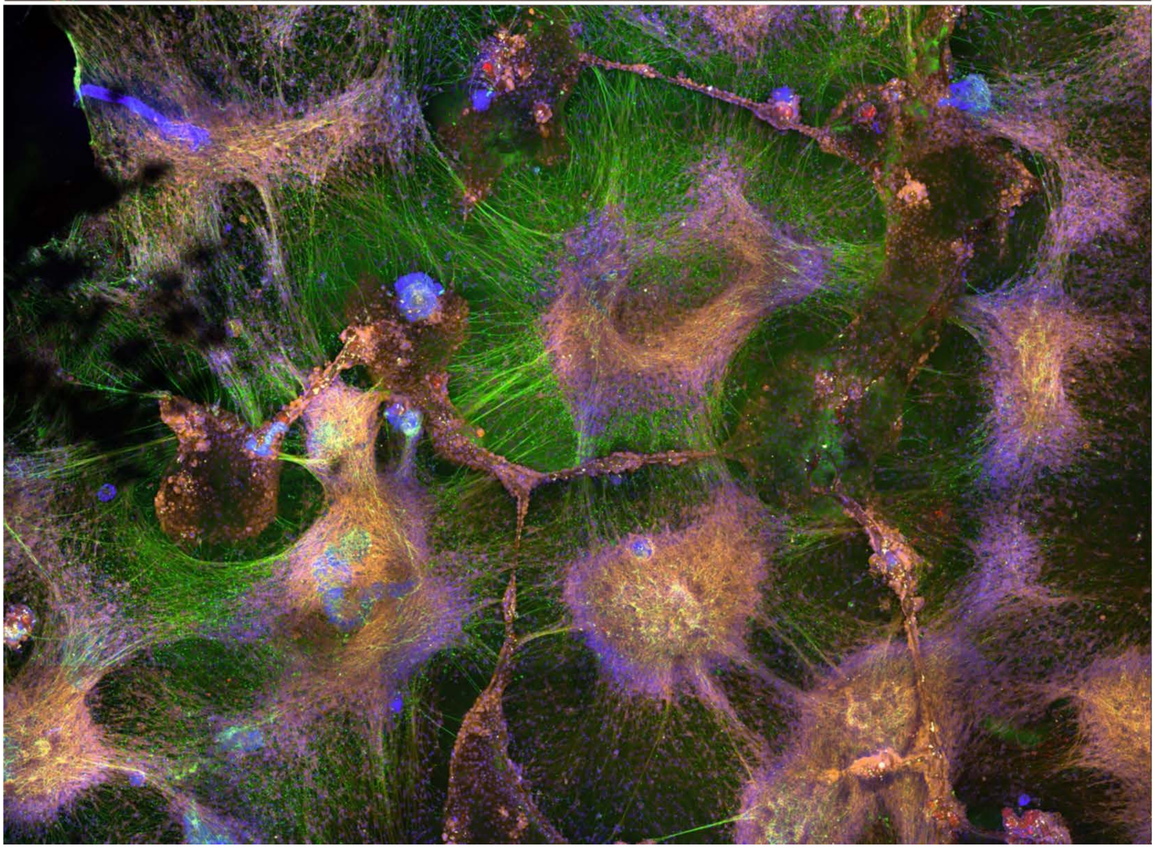

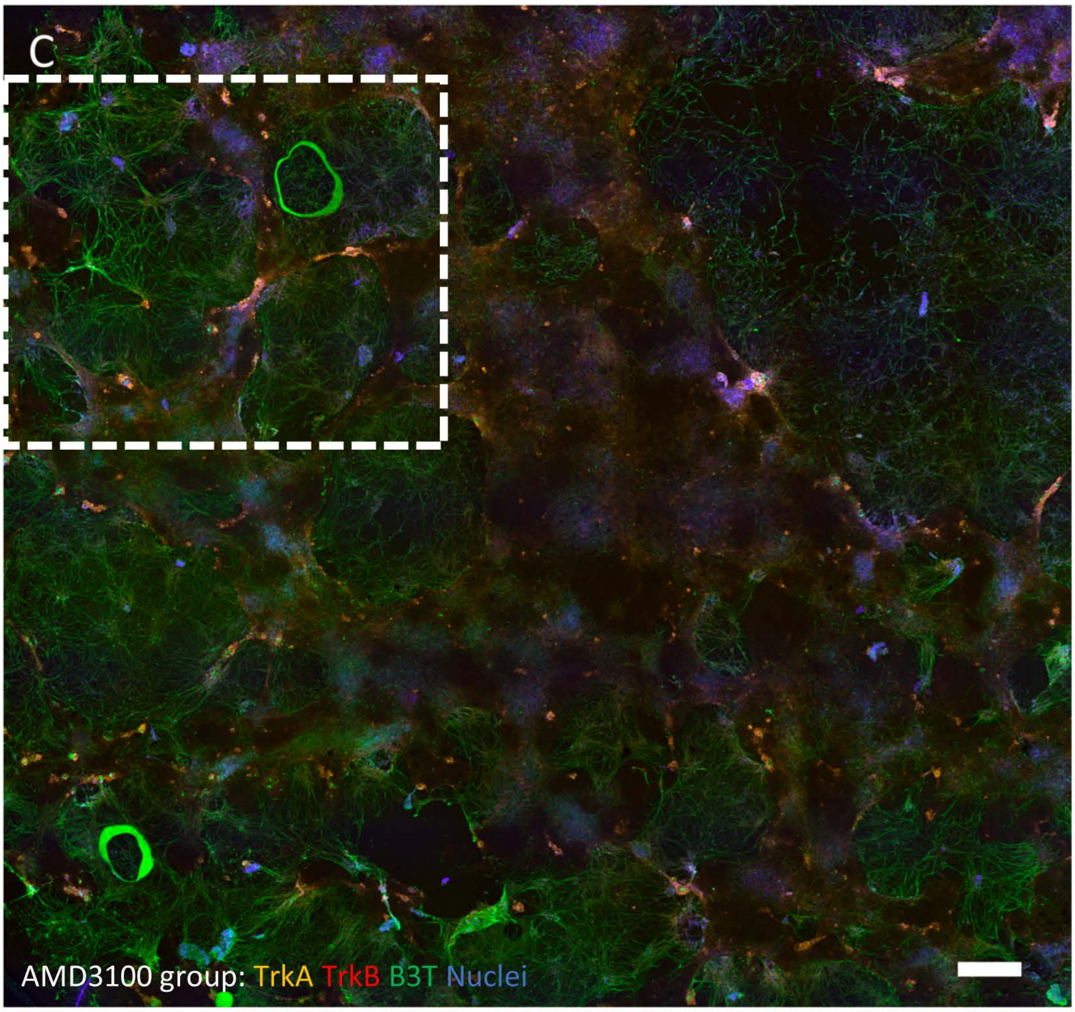

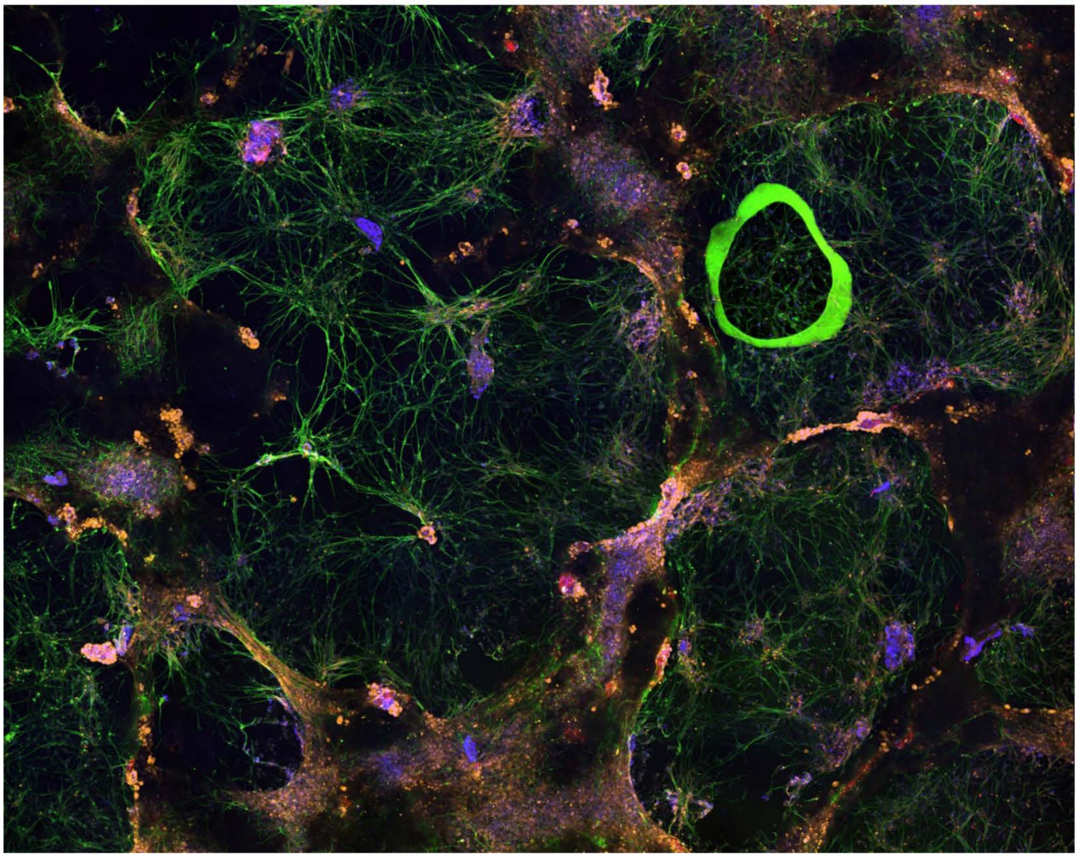

Figure S3 – Large-format image scans of cultures from the three differentiation groups. Upper image: 6x6 scan using the 10x objective, lower image: 6x4 scan of the outlined area using a 20x objective. A) Control B) SDF1 C) AMD3100 each stained against beta-III-tubulin (green), TrkA (yellow), TrkB (red) and nuclei (blue). See Fig. S4 for separate colour channels. Scale bars: 400µm

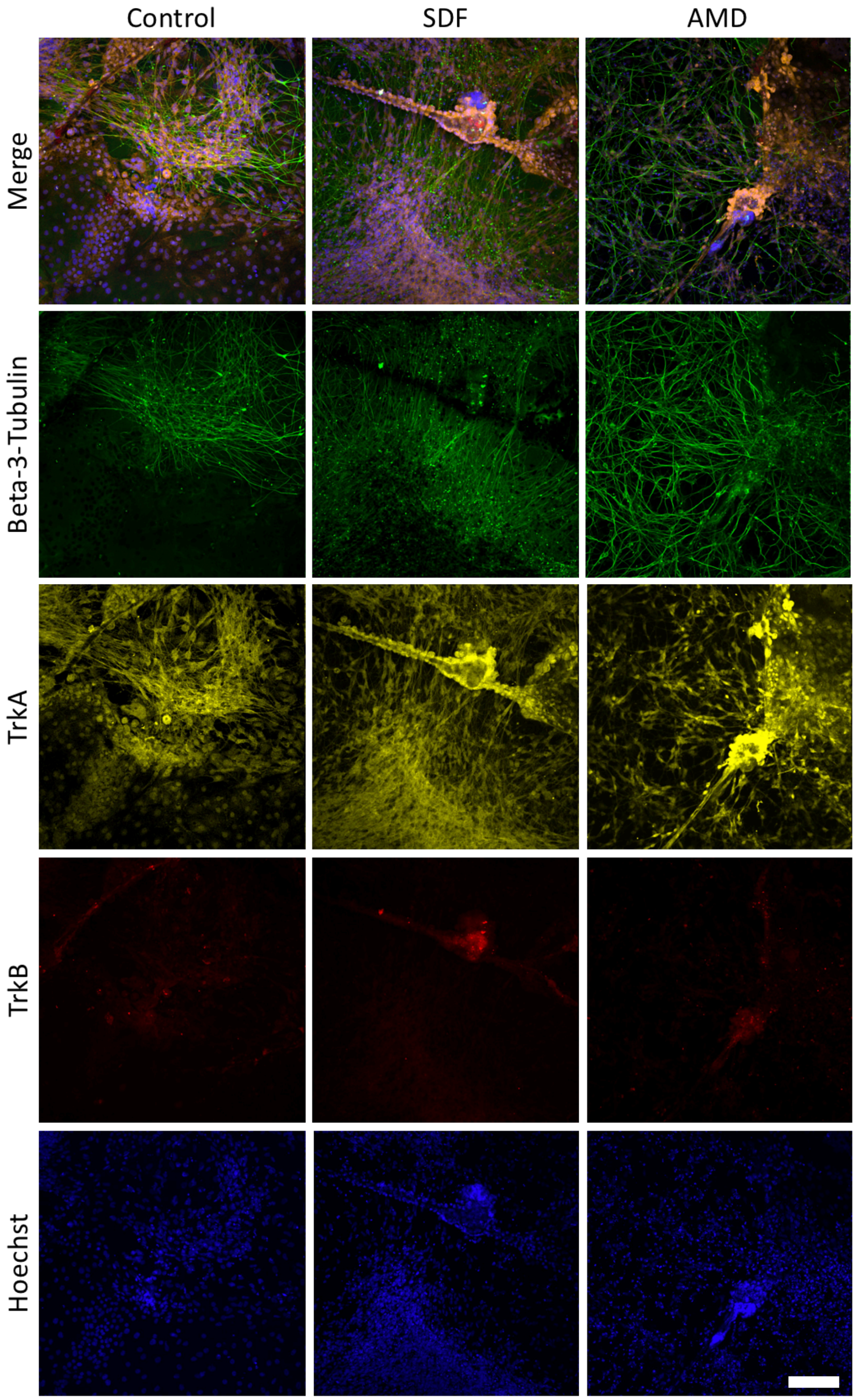

Figure S4 – shows individual colour channels of the three culture groups (control, SDF, and AMD) stained for beta-3-tubulin (green), TrkA (yellow), TrkB (red) and nuclear staining (blue). Scale bar: 150μm

Biology Open • Supplementary information

Table S1

Gene name	Primer sequence
GAPDH-F	ATGACATCAAGAAGGTGGTG
GAPDH-R	CATACCAGGAAATGAGCTTG
Otx2-F	GACCCGGTACCCAGACATC
Otx2-R	TGGCCACTTGTTCCACTCTC
Nanog-F	ATTCTTCCACCAGTCCCAAA
Nanog-R	ATCTGCTGGAGGCTGAGGTA
PAX7-F	GACGACGGCGAAAAGAAGG
PAX7-R	GTAGTGGGTCCTCTCAAAGGC
SOX10-F	CCTCACAGATCGCCTACACC
SOX10-R	CATATAGGAGAAGGCCGAGTAGA
TFAP2A-F	GACCTCTCGATCCACTCCTTAC
TFAP2A-R	GAGACGGCATTGCTGTTGGACT
FoxG1-F	AGAAGAACGGCAAGTACGAGA
FoxG1-R	TGTTGAGGGACAGATTGTGGC
Pax6-F	TCTTTGCTTGGGAAATCCG
Pax6-R	CTGCCCGTTCAACATCCTTAG
SOX7-F	ACGCCGAGCTCAGCAAGAT
SOX7-R	TCCACGTACGGCCTCTTCTG
PCDH1-F	GACTCTTCCAGATTGGGTCACAT
PCDH1-R	CTTGCCGCGGTCACTGA
Cad7-F	GCTACCCAGGTTGGGAATATC
Cad7-R	CTCTCCAAGTCTGTGTTTCTGT
Cad11-F	AGAGGTCCAATGTGGGAACG
Cad11-R	GGTTGTCCTTCGAGGATACTGT
L1CAM-F	GGCATTGAAATCCTCAACTCAAG
L1CAM-R	TATGTCTCTTGCTGTGCTTCC
TrkA-F	TTTGGCATGAGCAGGGATATC
TrkA-R	GTCGCTCTCGGTGGTGAACT
TrkB-F	ACTTCGTGCACCGCGATT
TrkB-R	CGAATGGGCAGCATTGTGT
CXCR4-F	AGCAGGTAGCAAAGTGACG
CXCR4-R	CCTCGGTGTAGTTATCTGAAGTG
P2X3-F	CCGCTGCGTGAACTACAG
P2X3-R	GTGAAGTTCTCAGCTTCCATCA
SDF1-F	GAGCCAACGTCAAGCATCTCA
SDF1-R	CGGGTCAATGCACACTTGTCTA
GFAP-F	CATGAAGCCGAAGAGTGGT
GFAP-R	TCGTTGGCTTCGTGCTT
NF-H-F	GACCTGCTCAACGTCAAGAT
NF-H-R	GGGAGTCCTTCAGTAAGAGAGA

Table S2

Antibody + origin	Product details + applied dilution in PBS
TrkA Rabbit	SAB1305370, Sigma-Aldrich, 1:100
TrkB Goat	T1941, Sigma-Aldrich, 1:100
Beta-III-tubulin mouse	T8660, Sigma-Aldrich, 1:2000
